# External variables influencing the attitudes of students toward AI acceptance in improving English writing: a systematic review

**DOI:** 10.3389/frai.2025.1719955

**Published:** 2026-01-21

**Authors:** Hafiza Sana Mansoor, Bambang Sumardjoko, Anam Sutopo

**Affiliations:** Faculty of Teacher Training and Education, Muhammadiyah University of Surakarta, Surakarta, Indonesia

**Keywords:** artificial intelligence, attitudes, constructivist learning theory, English writing skills, external variables, technology acceptance model

## Abstract

The aim of this systematic review is to examine and synthesize existing empirical evidence on external variables that influence students’ attitudes toward the acceptance of artificial intelligence (AI) in improving English writing skills. This research offers a conceptual framework, AI Constructivist Learning Model (AICLM), based on Technology Acceptance Model (TAM) and Constructivist Learning Theory (CLT). Motivation, engagement, and societal expectations, based on CLT, are identified as external variables in TAM. These three constructs support active, autonomous, and student-centered learning. A systematic search of academic databases was conducted following PRISMA (Preferred Reporting Items for Systematic Reviews and Meta-Analyses) guidelines. Sixteen empirical studies published from 2021 to 2025, indexed in Scopus, Web of Science, and Google Scholar, were included in this review. Articles were selected on the basis of certain keywords such as, AI, English writing, TAM, and CLT. Findings indicate that students perceive the ease of use and usefulness of AI if they have high motivation, more engagement, and positive societal expectations. Therefore, motivation, engagement, and societal expectations are significant external variables that influence the attitudes of students toward AI acceptance in improving English writing. AI integration in English writing development can be successful if the interaction between the constructs of TAM and CLT is understood well. CLT supports why and how students engage actively with AI tools. Students are more likely to accept AI if it increases motivation enhances engagement and fulfils societal expectations. This conceptual framework is significant for future researchers and teachers in designing effective AI-based writing instructional strategies and curricula.

## 1 Introduction

Many students struggle with English writing at the higher level, particularly in expressing complex ideas with accuracy, coherence, and appropriate academic style (Leli, 2020; Ramzan et al., 2023; Mansoor et al., 2025a,b,c). At university level, students require advanced skills beyond basic grammar and vocabulary, such as critical thinking, structured reasoning, academic style, and coherence (Jones and Hoffman, 1995). The limited use of AI tools can contribute to poor English proficiency among university students (Kot and Nykyporets, 2024; Mansoor et al., 2025a,b,c) because AI-powered applications, such as grammar checkers, paraphrasing tools, and writing evaluation tools, provide immediate, personalized, and regular support that conventional classroom instruction cannot offer. Artificial Intelligence (AI) in English as a Second Language (ESL) learning offers new opportunities of learning through instant feedback (AbuSahyon et al., 2023; Pokrivčáková, 2019) by replacing conventional language teaching such as lecture method, home task correction (Kannan and Munday, 2018; Tlili et al., 2021), pronunciation practice and writing evaluation (Florea and Radu, 2019; Shi et al., 2021). Recent developments in AI are influencing nearly every domain of life.

The development of tutoring systems, which helped in customizing lessons through computers in the 1960s, was the beginning of AI in education (Kelkar, 2022). Later on, the internet was used to assess the learners’ performance and to offer feedback through the development of expert systems (McCalla, 2023). AI prominently increased the accessibility to educational material through its implementation into online learning platforms in the 2000s (Guan et al., 2020). Now, AI has become advanced and flexible enough to meet individual needs through its customized features. For example, AI-assisted tools are enhancing pedagogy by improving assessment methodologies, providing personalized learning experiences to students (Adiguzel et al., 2023; Geldbach, 2023; Vorobyeva et al., 2025; Zou et al., 2020). Integration of AI in education, increases student engagement through customized learning, improves learning outcomes, and saves time (Xu, 2024). Despite the potential of AI to transform the learning environment fundamentally within English Language Teaching (ELT), AI implementation in ELT also raises ethical concerns, including the risk of cheating and the importance of balanced and responsible usage to confirm quality education for all learners (Ayala-Pazmiño, 2023; Balta, 2023).

Current literature mostly emphasizes the strategies and methods to integrate AI and technical development of AI systems (Divekar et al., 2021), with insufficient focus on the factors influencing AI acceptance in language learning settings (Zawacki-Richter et al., 2019). It is significant to review the attitudes of language learners and their actual use of technology (Blake, 2008). Attitudes, whether positive or negative, are essential to understand the intention of students toward technology as well as their future behaviours within the learning environment (Teo et al., 2006). It is the positive attitude of the users that makes the use of technology successful, no matter how sophisticated and powerful it is (Huang and Liaw, 2005; Rosen and Weil, 1995). According to Shadiev and Wang (2022), the variables that predict the acceptance and implementation of AI tools in language learning are important to enhance their effectiveness, increase adoption, and develop useful strategies. Moreover, this review provides a detailed understanding of language learners’ attitudes toward AI, along with the external variables influencing these attitudes (e.g., motivation, engagement, pedagogical context, and societal expectations), and helps technology developers, policymakers, and educators with insights to practical decisions. These insights can be used to guide AI tool development, curriculum design, teacher training, and institutional policy decisions related to AI-supported English writing instruction (Almushayt, 2022).

The Technology Acceptance Model (TAM), developed by Davis (1989), explains users’ acceptance of technology through four core constructs: Perceived Ease of Use (PEOU), Perceived Usefulness (PU), Attitude Toward Use (ATU), and Behavioral Intention (BI). A summary of these constructs and their definitions is provided in Table 1.

**Table 1:** Core constructs of the technology acceptance model (TAM).

Construct	Acronym	Definition
Perceived ease of use	PEOU	The degree to which a user believes that a system will be easy to use
Perceived usefulness	PU	The extent to which a user believes that using a system will be useful in performance
Attitude toward use	ATU	Users’ positive or negative view of using the system
Behavioral intention	BI	The strength of an individual’s intention to use a system, which predicts actual usage

According to Venkatesh and Bala (2008), explorative power of TAM can be restricted when it is used in isolation especially in complex learning environments such as language education, where emotional, social, and motivational dimensions also influence learners’ acceptance behavior. Consequently, this review identifies motivation, engagement, and societal expectations which are grounded in Constructivist Learning Theory (CLT) as external variables by extending TAM.

According to CLT (Vygotsky, 1978; Jonassen, 1999), knowledge is constructed actively when learners are involved in interaction, reflection, and engagement with their environment. Emotional engagement and intrinsic motivation of learners are vital to their academic success in the learning process. Students’ level of involvement and emotional connection with learning activities are reflected by engagement (Fredricks et al., 2004), while motivation helps them to indulge in learning activities and invest effort in developing writing skills (Deci and Ryan, 1985; Ushioda, 2011). Additionally, students’ attitudes toward technology use in writing are shaped by societal expectations, which include teacher beliefs, peer influence, and institutional norms (Huang and Liaw, 2005).

## 2 Literature review

### 2.1 TAM and CLT in English language learning

**Figure 1:**
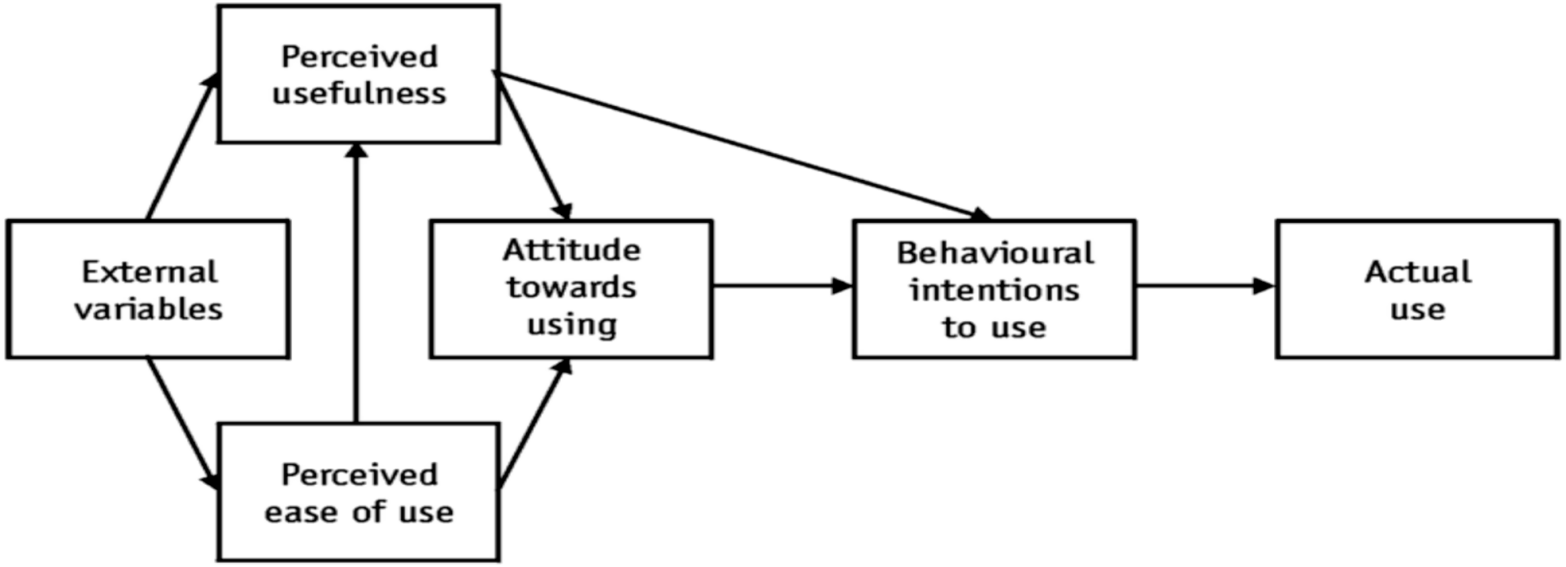
Technology acceptance model by Davis (1989).

The increased use of AI in education, and specifically in ESL writing, demands a comprehensive framework that facilitates the understanding of students’ attitudes influencing AI acceptance toward AI. Researchers argue that external contextual and psychological factors must be integrated to enhance TAM’s explanatory power, especially in complex learning scenarios such as writing in language education (Venkatesh and Bala, 2008; Alamer and Lee, 2021). This section synthesizes both the core and extended factors of TAM and CLT, along with the organized discussion of motivation, engagement, and societal expectation as external factors. TAM, originally developed by Davis (1989), is used in information systems and educational research (Teo and Noyes, 2011; Park, 2009). However, it has been criticized for lacking motivational and socio-cultural depth (Chiu et al., 2024). PU and PEOU are the key determinants of an individual’s attitude toward and intention to use technology. Subsequently, the TAM2 model, with subjective norms (social pressure) and facilitating conditions, was introduced by Venkatesh and Davis (2000) (see Figure 1).

Availability of smart devices, stable platforms, and access to high-speed internet significantly affect PEOU and consequently influence students’ intention to use AI. System characteristics can also act as external stimuli shaping PEOU as stated by Davis (1989). Additionally, in digital learning environments, poor infrastructure can negatively impact PEOU and reduce learners’ confidence in using AI tools (Alamer and Lee, 2021). Knowledge is actively constructed through reflection, social interaction, and authentic tasks (Jonassen, 1999; Vygotsky, 1978). Zone of Proximal Development (ZPD) by Vygotsky (1978) and scaffolding by Wood et al. (1976) are the main pillars of CLT. These concepts emphasize the effectiveness of guided learning through collaborative interaction and feedback, aligning with the real-time support of AI tools in writing tasks (Kohnke et al., 2023). Constructivist applications in second language writing highlight the role of learner autonomy, collaboration, and reflective practice (Nicol and Macfarlane-Dick, 2006; Herrington et al., 2010; Mansoor et al., 2025a,b,c). Previous research has highlighted different factors including contextual, environmental, and psychological factors that influence the acceptance of technology. Quality and availability of technological infrastructure is one of the most widely acknowledged external variables.

### 2.2 External variables and AI acceptance in English writing skills

One of the most significant external variables that impact students’ acceptance of AI tools is motivation. Self-Determination Theory (Deci and Ryan, 1985) presents two types of motivation: intrinsic and extrinsic. Intrinsic motivation is determined by the enjoyment and interest, on the other hand, extrinsic motivation is driven by the expectations of teachers and achievement. From the CLT perspective; meaningful engagement, autonomy, and goal-setting create motivation (Deci and Ryan, 2000). Research has shown that motivation affects PEOU as well as PU (Keller, 2008; Khan et al., 2025). Students are more likely to see AI tools as useful and easy to use if they perceive them as meaningful and aligned with their personal learning goals. As stated by Chiu et al. (2024), higher behavioral intentions to incorporate AI into writing practices are observed in motivated learners. Therefore, motivation not only aligns with CLT’s view of learners as active agents in their own development but also enhances TAM constructs.

Emotional, behavioral, and cognitive involvement in learning enhances engagement (Fredricks et al., 2004). According to Herrington et al. (2010), learners’ active participation is increased through personalized tasks, real-time feedback, and adaptive challenges when they use AI tools, which consequently support PEOU and PU. In digital learning environments, engagement plays the role of moderator and mediator in the acceptance of technology among students (Teo and Noyes, 2011). Engagement is significant for deep learning from a constructivist perspective. AI technologies are more likely to be accepted as they promise learner autonomy (Zimmerman, 2002) and support reflective practice (Nicol and Macfarlane-Dick, 2006). However, if the feedback is perceived as excessively general or lacks contextual relevance, AI systems may reduce engagement (Chapelle and Sauro, 2017).

Social pressures can directly influence the intention to use technology in educational settings as Venkatesh and Davis (2000) demonstrated by adding subjective norms into TAM2. Students’ intention to adopt unfamiliar and novel educational technologies is increased if they receive peer modeling and teacher support (Teo and Noyes, 2011). Therefore, attitudes and subjective norms toward adopting technology are shaped by the social influence such as reinforcement from family, teachers, and peers. According to Ifinedo (2017), university students increased PU of technology through organizational support. When AI in education is endorsed through national education policy, it improves the confidence of teachers and students prominently which positively influences PU and behavioral intention (UNESCO, 2021). Students’ perception of ease and readiness to use AI tools is affected by their prior training and digital literacy levels (Chiu et al., 2024). As mentioned by Park (2009), PEOU and PU in e-learning systems are influenced by prior experience with technology.

Students’ beliefs regarding autonomy, innovation, or conformity are also shaped by educational and cultural backgrounds. Li and Wang (2022) conducted research in China to explore the influence of communist cultural values on students’ intention to use AI writing tools. They found a strong influence of communist cultural values on students’ intention to use AI writing tools due to their alignment with societal expectations and teacher authority. Students’ attitudes and behavioral intention are also prominently affected by the concerns regarding data security, ethical use, and trust in AI. Lack of transparency and fear of surveillance reduce both PU and attitude toward use and stop users from sharing personal writing with AI platforms (Binns et al., 2018). AI tools that provide culturally and linguistically relevant feedback are favored by English language learners and also increase PU and intention to use (Alghamdi and Palaiologou, 2021).

Cultural norms, peer/teacher influence, and institutional pressures represent societal expectations which align with the subjective norm construct in TAM. Venkatesh and Davis (2000) highlighted that students’ intension to use technology is deeply influenced by their perceptions of how important the expectations of others are for them. The use of AI tools can be normalized and students’ behavioral intention can be increased if there is societal endorsement of AI tools through government policy, institutional support, or peer modeling (Fishbein and Ajzen, 1975; Li and Wang, 2022). Bandura (1986) presented the same idea of social learning in which students change their behavior toward learning according to the environment based on their feedback and observation. Societal influence helps in shaping learners’ attitudes, scaffolding mechanism, and identity development from the CLT perspective. Teacher-guided exploration and peer collaboration in the use of AI tools can increase students’ perceived competence and confidence, ultimately enhancing PU and behavioral intention.

### 2.3 Combination of TAM and CLT in understanding AI acceptance

CLT is based on social interaction, learner agency, and emotional engagement while TAM centers on behavioral intention theory and mainly focuses on cognitive appraisal of technology. However, both frameworks strive to recognize the perceptions of users regarding learning authenticity and usefulness. Motivation, social, and cultural dimensions of learning are the core constructs in CLT which directly increase PEOU and PU in TAM (Deci and Ryan, 2000; Davis, 1989). TAM is sometimes criticized for overlooking these constructs (Jonassen, 1999; Scherer, 2025). Constructivism focuses on collaborative and reflective learning (Mansoor et al., 2025a,b,c) while TAM emphasizes individual cognitive decision-making. There is a need to combine CLT and TAM to understand the influence of constructivist tools on the acceptance of AI by students in improving writing skills.

The integration of CLT into TAM assists in understanding the attitudes of students toward AI and supports the design of more learner-centered AI applications (Chiu et al., 2024; Mohammed and Kinyo, 2025). The aim of this systematic review is to analyze and identify constructivist-aligned variables that influence students’ attitudes toward AI in English writing and function as external factors in TAM.

GPT-based tools (Generative Pre-trained Transformer models), help in drafting support, grammar correction, and content expansion, have been widely used for idea generation in different empirical studies. The concerns about over-dependence and critical thinking remain, however, these studies highlight that these tools enhance writing fluency, motivation, and confidence, (Tram, 2025; Khan et al., 2025). Grammarly, a proprietary AI-powered writing assistant, shows strong links to perceived enjoyment, task relevance, and ease of use (Liang et al., 2024), as it has been used to support error detection, vocabulary improvement, and clarity revision. QuillBot, a proprietary AI-based paraphrasing tool, which assists with sentence restructuring and rewriting. However, some studies warn of excessive reliance on automated paraphrasing, it still increases students’ autonomy in revising drafts. Previous literature highlighted that Automated Writing Evaluation (AWE) systems such as Criterion and PEG Writing and Write & Improve (Cambridge English) enhance independent revisions and increase perceived usefulness. Such tools offer automated scoring and feedback on coherence, grammar, and organization. Academic writing processes are assisted by the use of machine translation tools (e.g., Google Translate) and multimodal platforms such as Blackboard Collaborate to assist academic writing processes (Yang and Liu, 2024; Alhumsi and Alshaye, 2021).

### 2.4 Summary of recently published review studies

Hu and Xiao (2025) highlighted the importance of personalization and support systems, focusing on the variables that affect online learning engagement such as learner and environmental factors. Khanfar et al. (2025) emphasized a holistic view for AI integration through categorization and examination of individual, social, organizational, environmental, and technological factors that influence the adoption of AI. Ma et al. (2025) emphasized the need for strategic implementation of ChatGPT and analyzed its adoption in higher education. Moreover, Zou et al. (2025) reviewed relevant literature to understand adoption in the context of Industry 4.0 through TAM and its extensions and argued that theoretical models are significant in identifying the dominant factors influencing technological acceptance (see Table 2).

**Table 2:** Previous systematic literature review (SLR) studies on the factors influencing the acceptance of AI.

Author(s)	Reviewed publication	Types and methods	Themes	Implications
Hu and Xiao (2025)	55 empirical studies published between January 2020 and July 2023	SLR, thematic analysis	Factors influencing online learning engagement by identifying key learner-related and environmental variables	Enhancing online engagement through personalization and support.
Khanfar et al. (2025)	90 peer-reviewed journal articles published between 2010 and mid-2022	SLR, Thematic content analysis	Key individual, social, organizational, environmental, and technological factors that influence the adoption of AI systems	Technological, organizational, environmental, and human-related factors should be considered for the successful AI adoption
Ma et al. (2025)	234 publications reviewed from November 30, 2022 to March 14, 2024	SLR, bibliometric analysis, thematic content analysis	Analyzes the research landscape of ChatGPT use and its adoption in higher education.	Guiding strategic integration of ChatGPT in higher education
Zou et al. (2025)	47 empirical studies published between 2003 and 2022	SLR, meta-analysis	Synthesizes empirical evidence on the adoption of industry 4.0 technologies using TAM and its extensions	Understanding key factors is essential to drive industry 4.0 adoption

This recent literature provides a clear understanding of different factors that influence AI acceptance; however, there is a lack of review of studies about the external factors that influence higher education students’ attitudes toward the acceptance of AI to improve their English writing skills. Moreover, there is a lack of specific models that combine CLT, which is effective in English language learning; with TAM that can help to explore AI related external factors in the context of English language learning. By reviewing empirical studies, this article aims to develop a conceptual framework by extending TAM and proposes a more comprehensive model to comprehend the acceptance of AI language learning tools. This review article primarily addresses the external factors, grounded in CLT, that influence students’ attitudes toward the acceptance of AI in improving English writing skills. The addressed research questions for this review are mentioned below:

1. What external variables influence students’ attitudes toward the acceptance of AI in improving English writing skills?
2. What key themes emerge from recent empirical studies (2021–2025) regarding students’ attitudes toward the acceptance of AI in English writing improvement?
3. How do CLT factors influence students’ attitudes toward accepting AI for improving their English writing skills through the TAM framework?

## 3 Methods

**Figure 2:**
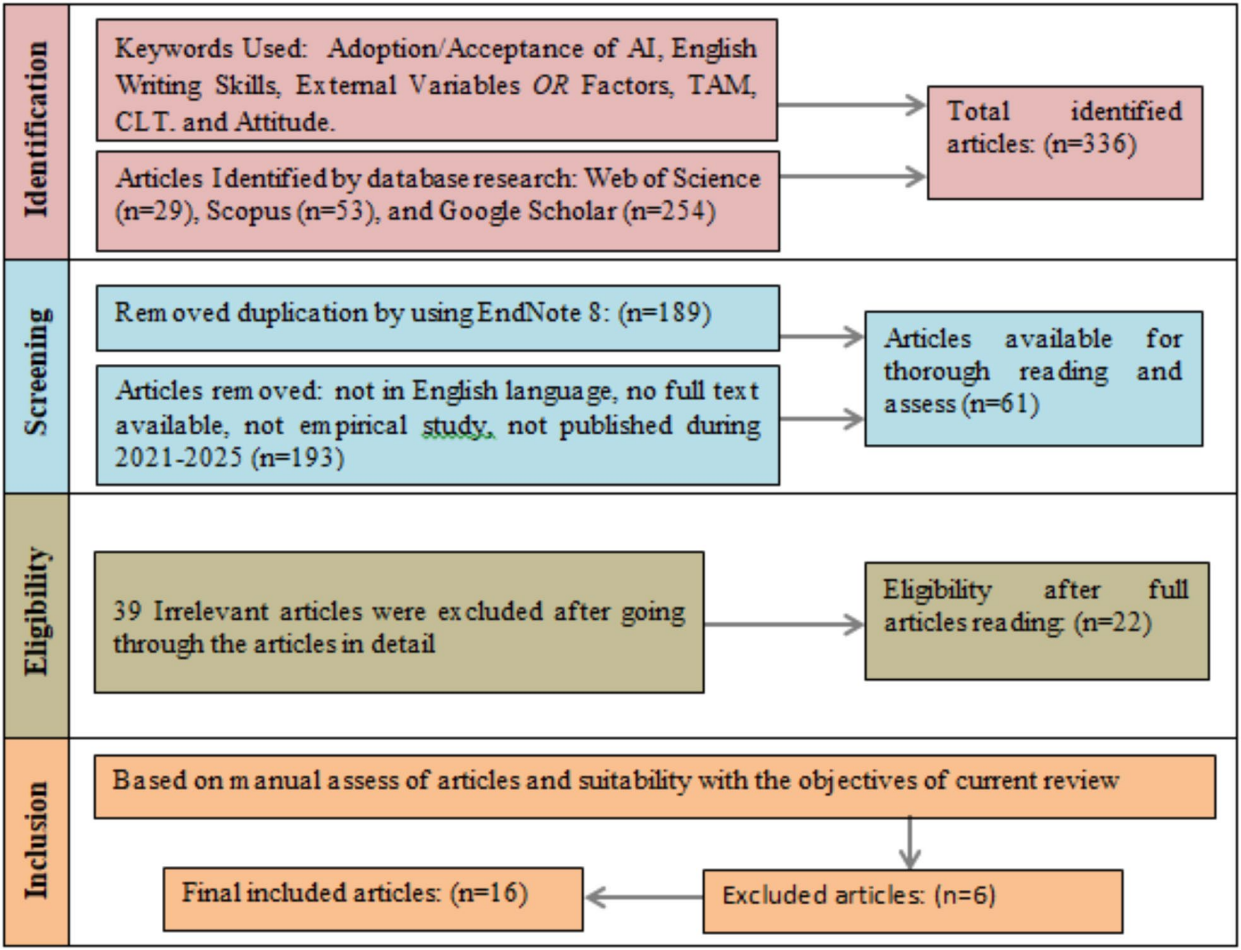
PRISMA flowchart of literature search process.

Preferred Reporting Items for Systematic Reviews and Meta-Analyses (PRISMA), as suggested by Moher et al. (2009), Liberati et al. (2009) guidelines are followed in this systematic review. PRISMA enhances the consistency and credibility of the research findings. It also assists scholars to conduct systematic reviews in a reliable, detailed, and clear manner. According to Nightingale (2009), the initial phase in conducting a SLR involves the formulation of a detailed protocol. This protocol should clearly state: (a) the main focus of the review; (b) the inclusion and exclusion criteria for the selection of relevant studies; (c) the method for the identification of studies; and (d) the approach to data analysis. In this protocol, the second step is the most significant as it determines the scope and overall findings of the study. A systematic process was used in this review to collect and analyze research about the use of AI in English writing skills. This process involved four basic steps such as literature retrieval, screening, content analysis, and bibliometric analysis. Sixteen peer-reviewed empirical studies published between 2021 and 2025 were selected for this review, with reference to AI grounded in TAM and English writing improvement based on CLT related constructs. PRISMA flowchart is given in Figure 2.

### 3.1 Identification

In the first phase of PRISMA, relevant articles are gathered through comprehensive and systematic database searches. In the first step, the researchers sought the articles, by using PICO (Population, Intervention, Comparison, Outcome) framework (Mamédio et al., 2009), related to “AI,” “English writing skills,” “TAM,” “CLT,” “external variables,” and “technology acceptance.” These keywords were sought in the abstract, title and keywords of the publication through Google Scholar, Web of Science, and Scopus databases. Scopus and Web of Science are the sources of inclusive, rigorous, high-quality academic literature search and Google Scholar increases access to grey literature and comprehensiveness. A summary of the search terms, databases, and their rationale is provided in Table 3.

**Table 3:** Literature search keywords and databases.

Item	Description	Rationale
Artificial intelligence/AI	Technologies and tools that enhance human intelligence, e.g., AI-based writing tools	Core concept underlying AI-assisted English writing
English writing skills	Academic and second-language writing performance and development	Focus of the educational outcome examined
Technology acceptance model/TAM	A theoretical framework explaining users’ acceptance of technology	Provides constructs for analyzing attitudes and acceptance
Constructivist learning theory/constructivism/CLT	Learning theory emphasizing active, social, and contextual learning	Explains motivational and engagement-related external variables
External variables/factors	Factors such as motivation, engagement, pedagogical context, access, and societal expectations	Influence attitudes toward AI adoption beyond system features
Technology acceptance/adoption	Users’ willingness to adopt and use technological tools	Outcome variable examined across studies

Searches were conducted in titles, abstracts, and keywords.

Twenty-nine records were identified from Web of Science, 253 articles from Google Scholar, and 53 from Scopus. At this step, 336 articles were identified between 2021 and 2025. This stage captures a broad range of studies related to the topic and confirms that the literature search is replicable, transparent, and comprehensive (Moher et al., 2009).

### 3.2 Screening

Three hundred thirty-six records were narrowed down to ensure the relevance of the studies in this phase. Many duplicate entries were found because multiple databases were searched. One hundred eight-nine articles were found duplicate and removed automatically. “Find Duplicates” function in EndNote 8 was used to match bibliographic information such as author, DOI, title, and year. This process ensured efficiency, accuracy, and consistency for record duplication. It is also useful in ensuring a transparent, documentable process and in decreasing human error, in accordance with PRISMA guidelines (Higgins et al., 2022). Furthermore, 193 articles were removed after the title and abstract screening because they were not in the English language, had no full text available, were not an empirical study, or were not published during 2021–2025. After manual screening of the title and abstract, 132 articles were not meeting the inclusion criteria. Therefore, 61 articles were selected for in-depth understanding and full-text reading.

### 3.3 Eligibility

In this phase, researchers carefully read the full text of all 61 records in detail which had already undergone the initial abstract and title screening, to determine the methodology and relevancy of the records. All the records were checked if they meet the research objectives (Page et al., 2021). Articles that involved populations other than university students and the use of AI in subjects other than English writing were excluded. Twenty-two empirical studies remained after applying all exclusion criteria. The initial search strategy captured articles from multiple global regions. However, no studies originating from European countries met the predefined inclusion criteria of this review. Some European publications were conceptual and focused on general AI or digital literacy rather than English writing skills. Some publications did not employ empirical methodology. The studies conducted outside the years 2021–2025 were excluded.

### 3.4 Inclusion

Twenty-two articles were read and evaluated thoroughly at this stage by the researchers to ensure methodological rigor and theoretical relevance to the present review. Two professors, one from an Indonesian university, an expert in English language teaching and the second, from a Pakistani university, an expert in educational technologies, evaluated the full-text articles based on predetermined quality criteria. Evaluation results from both experts were compared and Cohen’s Kappa coefficient was used to measure the degree of agreement. Cohen’s Kappa coefficient is an extensively renowned statistical method for assessing inter-rater reliability (McHugh, 2012). Six articles were excluded during the detailed assessment because of a different population, a different theoretical framework (Higgins et al., 2022), and findings not clearly stated. Finally, 16 articles were selected that represented the most important and theoretically strong studies to support the objectives and conclusions of the current review. The analysis, conducted using SPSS 23, produced a Kappa score of 0.84, which indicates a “strong agreement” as defined according to the interpretation scale by Landis and Koch (1977). Therefore, the validity and reliability of the record selection process are reflected by the high level of agreement, which strengthens the findings.

## 4 Results and discussion

**Figure 3:**
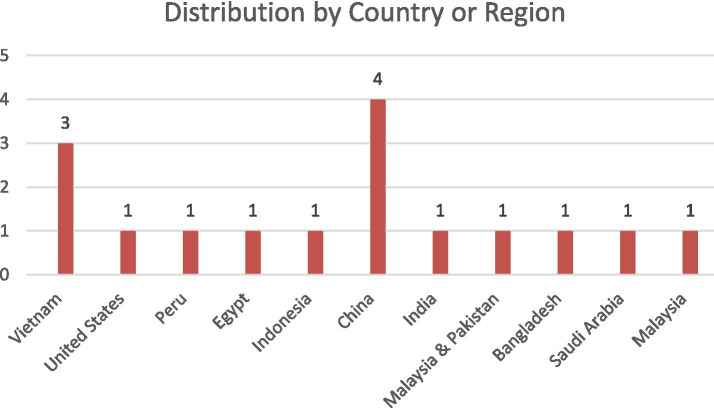
Geographical distribution of literature.

The reviewed articles were published in Asia, the Middle East, North America, and South America, which show geographically diverse scholarly interest in AI-assisted English writing. China contributed the highest number of publications (*n* = 4), followed by Vietnam (*n* = 3), while the remaining each country (Malaysia, the United States, Saudi Arabia, Peru, Bangladesh, Egypt, Pakistan, India, and Indonesia) contributed in one study, as shown in Figure 3.

**Figure 4:**
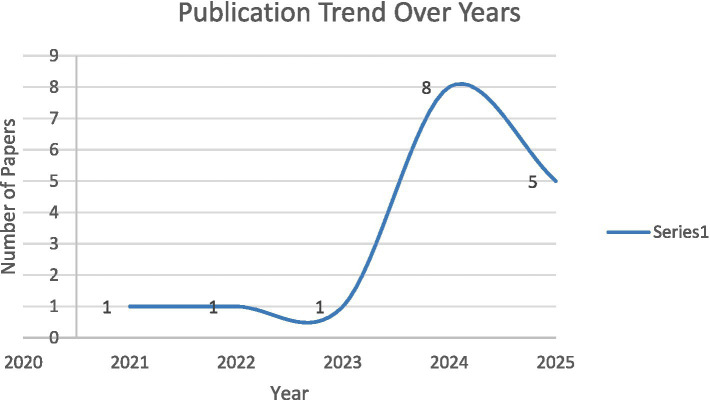
Publication trend over the years.

The number of studies remained low between 2021 and 2023, with one article published in each year, followed by an increase in 2024. A smaller number of studies were identified in 2025 at the time of data collection. Given the limited number of included articles and the timing of the review, these publication patterns should be interpreted cautiously and are presented to contextualize the reviewed literature rather than to indicate impact, effectiveness, or adoption levels. Selected peer-reviewed research publications from 2021 to 2025 are shown in Figure 4.

### 4.1 RQ1: external variables influence students’ attitudes toward the acceptance of AI

Sixteen peer-reviewed studies were critically reviewed, highlighted the trend of AI acceptance among university students for English writing assistance. This review revealed a consistent pattern indicating that TAM needs an extension through the lens of CLT. Emerging findings suggest that motivation, engagement, societal expectations, and constructivist learning strategies are essential in determining students’ attitudes toward AI, especially in English language learning, while core constructs of TAM such as PU, PEOU, ATU, and BI remain functional. External variables that influence students’ attitudes toward AI acceptance in enhancing English writing skills are identified in these 16 peer-reviewed articles published between 2021 and 2025. TAM is used in all selected articles, in combination with other theoretical frameworks such as the Unified Theory of Acceptance and Use of Technology (UTAUT), Social Cognitive Theory (SCT), and Cognitive Load Theory (CLT). These frameworks helped researchers explore various external factors that influence students’ attitudes towards accepting AI. The data of 16 peer-reviewed articles is given below (see Table 4).

**Table 4:** Selected peer-reviewed research data (2021–2025).

No.	Author(s)/country	AI Tool/context	TAM external variables	Key findings	CLT variables
1	Tram (2025), Vietnam	ChatGPT use for academic writing	Performance expectancy, effort expectancy, social influence, facilitating condition	All four variables significantly influence ChatGPT use but performance expectancy is the strongest predictor	Motivation, societal expectations, learning strategies
2	Salam (2025), Indonesia	ChatGPT for writing assignments	Not explicitly stated	Ease of use influenced usefulness and attitude; usefulness influenced attitude; attitude predicted intention to use. Students accepted ChatGPT mainly as a writing aid.	
3	Mahfouz and AbdelMohsen (2025), Egypt	ChatGPT use in writing language essays	Ethical appropriateness, higher-order thinking	Students held positive attitudes toward ChatGPT’s usefulness and ethics, but voiced concerns about its impact on critical thinking; institutional support was favored.	Societal expectations
4	Wang (2025), China	Large language models for academic writing	Performance expectancy, social influence	Performance expectancy and social influence strongly influenced behavioral intention; motivation was a key predictor of both intention and actual use.	Motivation, societal expectations
5	Khan et al. (2025), India	ChatGPT in academic writing	Learner confidence, cognitive engagement	ChatGPT improved fluency, motivation, and reduced anxiety; concerns included over-reliance, reduced critical thinking, and integrity issues; recommended structured AI integration and AI literacy	Engagement
6	Acosta-Enriquez et al. (2024), Peru	ChatGPT use in academic activities	Responsible use, intention to use, acceptance, positive emotions, risk, boredom, information verification	Responsible use, frequent intention to use, and acceptance strongly predict positive attitudes; risk and boredom negatively influence attitude; verifying information supports responsible use.	Motivation, engagement, societal expectations
7	Liang et al. (2024), China	Grammarly for English writing	Perceived enjoyment, task relevance, subjective norm	Ease of use influenced usefulness and intention; enjoyment and task relevance were strong predictors; some TAM paths (PU → ATU, SN → PU) were not supported.	Motivation, societal expectations
8	Farooq M. et al. (2024) and Farooq S. et al. (2024), Malaysia & Pakistan	AI writing tools for sustainable academic writing	Ethical considerations, sustainability in writing	Perceived usefulness and ease of use strongly influenced attitudes and intention; utility valued over barriers; ethical use is critical for sustainable writing.	Societal expectations
9	Awal and Haque (2025), Bangladesh	ChatGPT, Google Bard in higher education	Time-saving feature (TSF), academic self-efficacy (ASE), electronic word-of-mouth (EWOM)	TSF, ASE, and EWOM positively influence intention to adopt; intention significantly affects actual use; PEOU negatively impacts intention.	Societal expectations
10	Yang and Liu (2024), China	Machine Translation (MT) in academic reading and writing	Knowledge of MT, positive evaluation	Students positively perceive and scientifically use MT; it improves academic English and supports MT literacy development	Motivation, learning strategies
11	Abd Hadi et al. (2024), Malaysia	ChatGPT in academic writing	Attitude, motivation	Students showed strong acceptance of ChatGPT; attitude was the strongest predictor of usage.	Motivation
12	Ge (2024), China	ChatGPT in English writing education	Utility, effectiveness, user satisfaction, privacy concerns	Students showed positive acceptance of ChatGPT but still valued educators’ roles and expressed privacy concerns.	Motivation, societal expectations
13	Nguyen and Dieu (2024), Vietnam	ChatGPT for writing assistance	Familiarity, usage purpose, challenges	Students had positive perceptions and frequent use for academic writing; faced challenges; suggested improvements for better use.	Motivation, engagement
14	Thao and Thuy (2023), Vietnam	ChatGPT in EFL writing tasks	Engagement, learner autonomy, social influence, ethical concerns	ChatGPT enhanced engagement and writing skills but raised issues of over-reliance, accuracy, and ethics.	Engagement, societal expectations
15	Choung et al. (2022), United States	AI voice assistants and smart technologies	Trust (human-like trust & functionality trust)	Trust significantly influences perceived usefulness and attitude, which in turn affect behavioral intention. Functionality trust has a greater total effect than human-like trust in AI acceptance.	Societal expectations
16	Alhumsi and Alshaye (2021), Saudi Arabia	Blackboard collaborate for academic writing	Perception	Positive perceptions; attitude toward use is the strongest predictor of intention to use blackboard.	

This review highlighted that students’ attitudes toward the acceptance of AI in improving English writing skills are strongly influenced by four external variables: motivation, engagement, societal expectations, and learning strategies. Although these studies were conducted in different contexts, they converge on some key findings. Studies conducted by Tram (2025), Salam (2025), Ge (2024), and Abd Hadi et al. (2024) employed the TAM framework to explore AI acceptance to support English writing skills. PU is identified as consistently central determinant, confirming that if students perceive AI tools as effective in learning, they are more likely to accept it. Furthermore, motivation as a significant external variable is directly linked in shaping the behavioral intentions of the students (Wang, 2025; Khan et al., 2025; Liang et al., 2024). Privacy (Ge, 2024) and trust (Choung et al., 2022) about AI use require strong policy frameworks and institutional guidelines.

### 4.2 Motivation

Motivation, encompassing both intrinsic interest and extrinsic rewards in learning contexts, refers to students’ internal drive to adopt AI for English writing. It is a process that prompts, directs, and sustains goal-oriented behavior (Deci and Ryan, 2000; Schunk et al., 2014). Performance expectancy, perceived usefulness, time-saving benefits, and academic self-efficacy are related to motivation either intrinsic or extrinsic and function as a consistent positive predictor of attitude and behavioral intention in TAM (Tram, 2025; Wang, 2025; Awal and Haque, 2025; Abd Hadi et al., 2024; Khan et al., 2025). Motivation plays a key role in students’ acceptance of AI tools, as revealed through empirical studies.

Wang (2025) identified motivation as a primary predictor of both BI and actual AI use by employing an extended UTAUT model. Emotional participation, cognitive engagement, and enjoyment in using AI tools enhance student motivation (Khan et al., 2025; Liang et al., 2024). According to Mahfouz and AbdelMohsen (2025), students have a positive attitude toward AI not only because of PEOU and PU, but also because they accept AI from ethical perspectives to improve their English. Similarly, perceived enjoyment, which is a factor of motivation, was used by Liang et al. (2024) as an external variable in TAM to understand students’ intention to use Grammarly. It supports the need to integrate self-determined and affective variables beyond TAM’s conventional constructs. These findings reinforce the importance of incorporating both intrinsic and extrinsic motivation within an extended TAM framework.

### 4.3 Engagement

Engagement refers to the cognitive, emotional, and behavioral involvement of students in AI-assisted English writing tasks, reflected in enjoyment, task relevance, autonomy, and sustained interaction with the tool (Liang et al., 2024; Thao and Thuy, 2023; Khan et al., 2025). Moreover, engagement includes learners’ active participation in learning activities, which increases PU and promotes deeper learning outcomes (Fredricks et al., 2004; Reeve, 2012). Engagement influence the attitudes of students positively through enjoyment, task relevance, and autonomy, higher PEOU and PU (Liang et al., 2024; Thao and Thuy, 2023; Khan et al., 2025). Engagement (Khan et al., 2025), expectations of performance benefits (Wang, 2025; Tram, 2025), social pressures (Wang, 2025; Tram, 2025), and ethical dilemmas (Thao and Thuy, 2023; Farooq M. et al., 2024; Farooq S. et al., 2024) are among the most frequently examined external variables as explored by previous researchers.

Cognitive engagement and learning strategies also have been shown to shape students’ attitudes. Students significantly increase their engagement if they use ChatGPT for creating ideas, learning coherence, brainstorming, and improving grammar (Thao and Thuy, 2023; Salam, 2025). These activities increase learners’ interaction with content and autonomy, as engagement is a vital factor in CLT, which influence AI acceptance. A qualitative study with Indian EFL learners conducted by Khan et al. (2025) affirmed that sustained engagement with AI tools increase confidence and writing fluency. Increased engagement makes AI use insightful and focused if does not lead to over-reliance. The inclusion of engagement as an external variable in TAM is supported by these findings, as it serves both as a facilitator and an agent in AI acceptance.

### 4.4 Societal expectations

Societal expectations refer to the perceived social norms, institutional policies, and peer or instructor influences that shape the attitudes and acceptability of using AI tools for English writing (Tram, 2025; Ge, 2024; Awal and Haque, 2025). Moreover, it is related to social influence where positive reinforcement, institutional help, and social status of AI increase its adoption. However, policy restrictions or negative norms may hinder acceptance of AI (Venkatesh et al., 2003; Liang et al., 2024). Social influence such as the AI endorsement by parents, teachers and peers also influence its adoption by students (Awal and Haque, 2025; Nguyen and Dieu, 2024). Moreover, external variables such as interface design of AI, digital literacy, and familiarity with tools are significant, as students’ confidence and prior experiences also affect their attitudes toward AI (Ge, 2024; Yang and Liu, 2024). Social influence, electronic word-of-mouth, and institutional support enhance the use of AI and increase its acceptance (Tram, 2025; Ge, 2024; Awal and Haque, 2025; Liang et al., 2024).

Social influence is another dominant factors identified in the literature. Institutional and cultural contexts impact students’ decisions to adopt AI writing assistants, as identified in previous research. Moreover, the role of electronic word-of-mouth (EWOM), social influence, and peer dynamics is underscored by Awal and Haque (2025) and Tram (2025). According to them, social influences shape students’ behavioral intention toward the use of AI tools. Societal and institutional standards significantly affect the sustainable and ethical use of AI in academic writing, as highlighted by Farooq M. et al. (2024), Farooq S. et al. (2024) and Ge (2024). According to Choung et al. (2022), trust facilitates PU and BI and is further elaborated into two dimensions: human-like trust and functionality trust. According to these findings, societal expectations may help explain students’ acceptance of AI in writing assignments, as they are linked to institutional endorsement, peer influence, and cultural context.

### 4.5 Learning strategies

Learning strategies are deliberate cognitive, metacognitive, and resource-management techniques employed by students to process, organize, and retain information to enhance understanding and performance (Oxford, 2011; Weinstein and Mayer, 1986). Students use some purposeful approaches when engaging with AI tools, such as aligning tasks to learning goals, verifying information, fostering higher-order thinking, and promoting autonomy to improve English writing (Yang and Liu, 2024; Thao and Thuy, 2023; Mahfouz and AbdelMohsen, 2025). Nguyen and Dieu (2024) and Abd Hadi et al. (2024) elaborated on strategies, such as generating ideas, brainstorming, organizing ideas, drafting content, and correcting grammar, which were employed by students while using ChatGPT. These strategies are directly linked with constructivist learning strategies such as self-reflection, scaffolding, and interactive feedback.

ChatGPT provides students with contextual, learner-driven, and active experiences, which lead to higher acceptance. Yang and Liu (2024) suggested that teachers should train students in the appropriate use of AI. They also stressed the purposeful and planned use of Machine Translation (MT) tools to improve students’ writing and reading skills. AI tools can be pedagogically integrated to develop metacognition, deeper cognitive engagement, and ethical reasoning. However, Ethical concerns, such as lack of critical thinking, over-dependency, and plagiarism, significantly impact students’ acceptance of AI (Thao and Thuy, 2023; Mahfouz and AbdelMohsen, 2025). CLT views student as active agent of knowledge who engage with these pedagogical techniques. Evidence supports integrating these four external variables into a modified TAM, as they consistently influence attitudes toward AI use in English writing through their impact on PU, PEOU, and social acceptability, aligning with principles of CLT.

### 4.6 RQ2: visualization of key themes in selected literature

**Figure 5:**
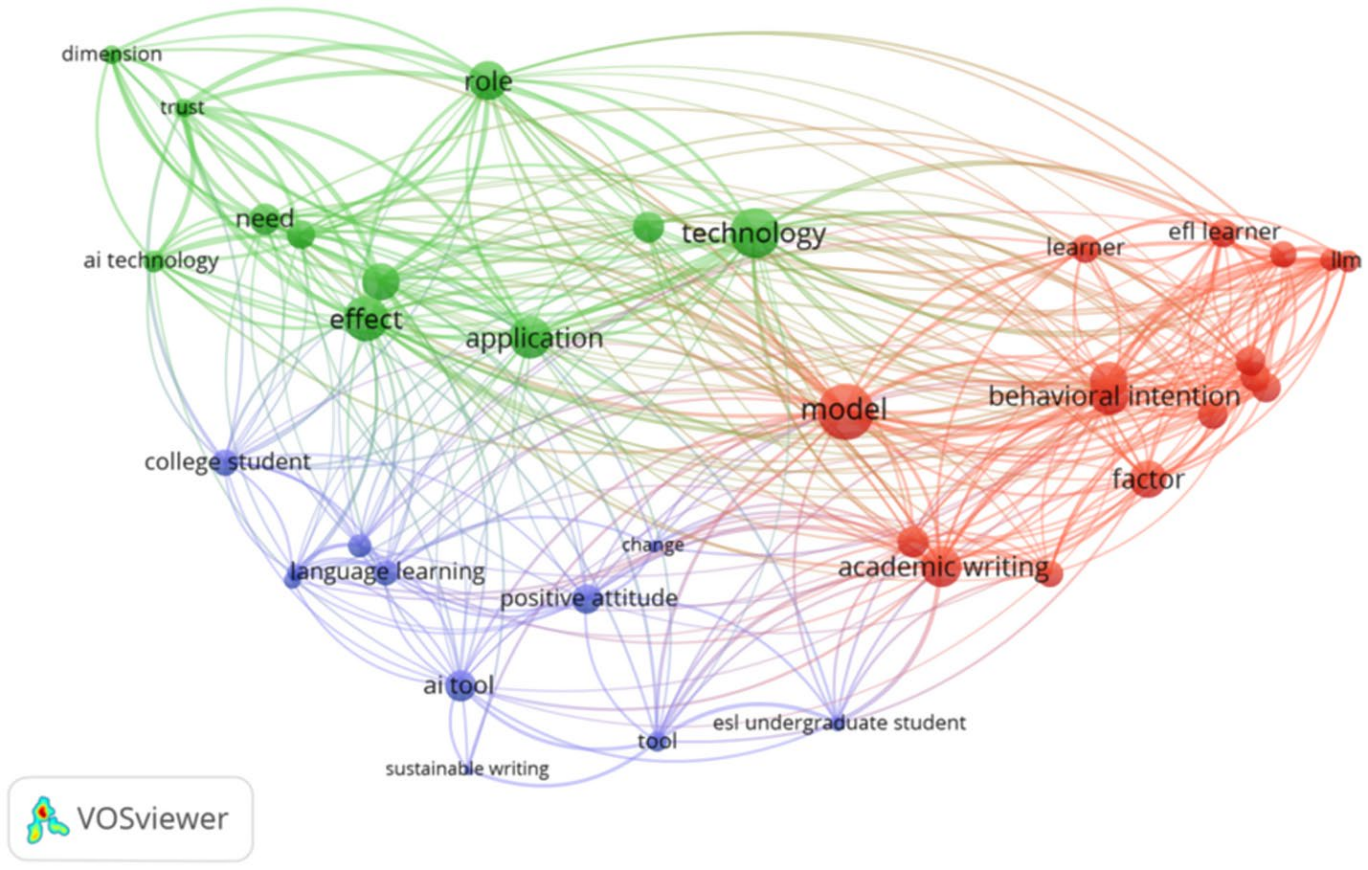
Co-occurrence network of keywords.

VOSviewer is a bibliometric visualization software that maps and clusters relationships among research items, such as authors, documents, or keywords on the basis of their co-occurrence or citation links. In this review, a co-occurrence analysis of keywords was conducted using VOSviewer to visually explore patterns in the literature based on 16 peer-reviewed articles published from 2021 to 2025. Figure 5 of the network map shows three main clusters, represented by red, purple, and green colors. This process allowed the occurrence of distinct clusters representing motivation, engagement, societal expectations, and learning strategies.

The software applies mapping techniques and clustering algorithms (e.g., modularity-based clustering) to group frequently co-occurring keywords into themes, with the proximity and size of nodes indicating their relevance and interconnections (Van Eck and Waltman, 2010). This approach provides an objective, data-driven basis for theme development, reducing researcher bias and enabling the identification of conceptual links that may not be directly visible from manual coding alone (Donthu et al., 2021; Perianes-Rodriguez et al., 2016). Using VOSviewer in this study supported the methodological accuracy by visualizing the logical structure of the previous research. This method associates the thematic synthesis with strong bibliometric evidence, which align with the best practices in systematic reviews that integrate both qualitative and quantitative analysis. Meaningful thematic groups are formed by the frequently co-occurring terms are indicated in each cluster. This clustering explores underlying patterns, which lead to the development of a modified model AI Constructivist Learning Model (AICLM) based on TAM and CLT.

The red cluster, with the theme of *Learner Motivation and Theoretical Models of AI Acceptance*, is directly linked to the motivation construct, as it emphasizes performance expectancy, PU, and BI, which are core drivers that stimulate learners’ willingness to adopt AI for English writing improvement (Tram, 2025; Wang, 2025; Abd Hadi et al., 2024). Societal expectations also appear here through social influence and the role of institutional norms, which influence motivation. This theme focuses on theoretical underpinnings, learner behavior, and motivation-related variables. These keywords are dominant within TAM and its extensions, such as UTAUT. Some psychological constructs, such as performance expectancy, behavioral intention, social influence, and model directly influence AI acceptance. There is a strong interaction between theoretical models and individual learner characteristics, including EFL learner and motivation. Keywords in red area are significant in determining student intention to use AI tools in non-native English contexts for writing enhancement (see Table 5).

**Table 5:** Clusters of keywords with themes.

Areas	Keywords	Major themes
Red area	Academic writing, behavioral intention, EFL learner, English academic writing, factor, learner, model, motivation, performance expectancy, quantitative approach, relationship, social influence, unified theory, university, UTAUT	Learner motivation and theoretical models of AI acceptance
Purple areas	AI tools, change, college student, concern, ESL undergraduate student, language learning, limitation, positive attitude, stakeholder, sustainable writing, tool	Student engagement, learning experiences, and contextual factors
Green area	AI technology, application, dimension, effect, intention, need, paper, path analysis, role, technology, trust	Technological influence, trust, and constructivist dimensions

The purple cluster, with the theme of *Student Engagement, Learning Experiences, and Contextual Factors* matches the engagement construct by covering affective (enjoyment, positive attitude), cognitive (task relevance, autonomy), and behavioral (active use) dimensions that sustain students’ involvement with AI tools (Liang et al., 2024; Thao and Thuy, 2023; Khan et al., 2025). This cluster also points at societal expectations by highlighting the idea of stakeholder involvement and the influence of institutional contexts on learner experiences. This theme highlights the real-world and practical aspects of the use of AI tools by language learners. Terms such as change, positive attitude, and language learning represent the behavioral and affective aspects of students. Additionally, keywords such as limitation and sustainable writing reflect challenges and perceived affordances faced by students in English writing learning. Societal and institutional influences on the acceptance of AI are hinted by the involvement of stakeholders.

The green cluster, *Technological Influence, Trust, and Constructivist Dimensions*, connects with the learning strategies construct, which highlights trust, information verification, higher-order thinking, and the pedagogical integration of AI as a scaffold for active learning (Mahfouz and AbdelMohsen, 2025; Yang and Liu, 2024). In this cluster, trust and ethical considerations also overlap with societal expectations because institutional policies and norms may influence AI acceptance. Collectively, all three themes reflect students’ attitudes toward AI in English writing are influences by motivation, engagement, societal expectations, and learning strategies. These variables directly influence, either positively or negatively, PEOU, PU, and attitudes which lead to BI and actual use of AI. This visualization justifies their integration as external variables in the modified TAM, grounded in CLT.

### 4.7 RQ3. CLT constructs function as external variables within the TAM

**Figure 6:**
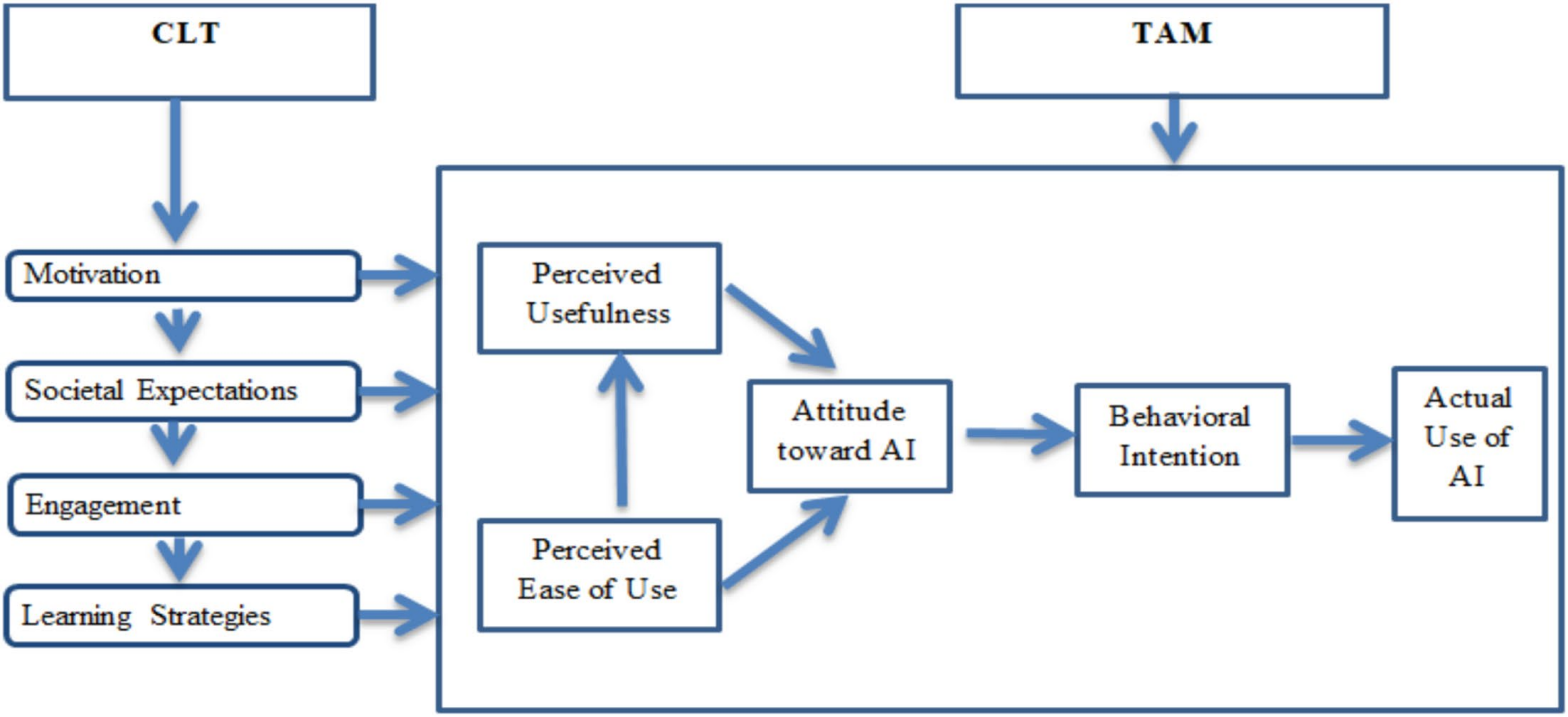
AI constructivist learning model (AICLM).

Previous researchers combined different theoretical frameworks and external variables with TAM to explore the acceptance of AI. Awal and Haque (2025) combined TAM with Social Cognitive Theory (SCT) and illustrated that, in some cases; PEOU and PU are weaker indicators than academic self-efficacy. Similarly, students’ positive attitudes are shaped by culturally and pedagogically relevant learning experiences (Alsaedi and Alhumsi, 2024; Salam, 2025). Therefore, while traditional TAM is effective in explaining initial technology adoption in general education, it is deficient in elaborating socio-cognitive and motivational dimensions that are significant for AI integration in English writing development. AICLM, combining TAM and CLT, based on recently published review articles and their systematic analysis, is presented below (see Figure 6).

TAM has limitations in educational contexts regardless of its robust framework. It ignores the full complexity of human behavior such as how people really think and act (Chiu et al., 2024). Social and affective aspects such as motivation, engagement, and societal expectations that influence learners’ technology adoption, particularly in language learning, are overlooked by TAM. Similarly, CLT lacks predictive models for technology use behavior while it explains the processes of deep learning only. TAM is helpful as an empirical structure for analyzing behavioral intention; on the contrary, CLT provides rich qualitative insights. The lack of combined models that integrate the predictive strength of TAM with the pedagogical richness of CLT is found as a key gap in the literature.

Based on the review of the literature, there is a need to extend TAM while adding external variables from CLT which affect the attitudes of students toward the acceptance of AI in developing English writing skills. Growing body of research and the existing reviewed literature highlights various factors influencing the adoption and acceptance of AI in educational domain. The reviewed literature generally supports an expanded TAM that incorporates CLT-based external variables such as motivation, engagement, societal expectations, and constructivist learning strategies. Across the reviewed studies, learners’ acceptance of AI tools is consistently related to “perceived usefulness,” “ease of use,” and “positive attitudes,” which were shown to predict “intention to use” AI for academic writing (Salam, 2025; Abd Hadi et al., 2024; Alhumsi and Alshaye, 2021). Several studies further emphasize motivational and engagement-related factors, reporting that AI tools improved “motivation,” “engagement,” and writing fluency, while reducing anxiety (Khan et al., 2025; Thao and Thuy, 2023). Furthermore, societal and contextual influences were frequently highlighted, with studies investigating the importance of “social influence,” “ethical considerations,” and “responsible use” in strengthening students’ attitudes toward AI adoption (Mahfouz and AbdelMohsen, 2025; Acosta-Enriquez et al., 2024; Farooq M. et al., 2024; Farooq S. et al., 2024). Based on these empirical findings, the proposed AI Constructivist Learning Model (AICLM) seeks to comprehensively explain AI acceptance in English academic writing and may inform effective and responsible teaching practices for AI-supported writing instruction.

In AICLM, four CLT derived constructs, such as motivation, engagement, societal expectations, and learning strategies, act as external variables which influence students’ cognitive beliefs about technology, particularly PU and PEOU, ATU and BI. Motivation, including performance expectancy, time-saving benefits, and academic self-efficacy, particularly increases PU and can directly enhance BI when self-efficacy is high (Tram, 2025; Wang, 2025; Awal and Haque, 2025; Khan et al., 2025). Engagement, through enjoyment, task relevance, and autonomy, reinforces both PU and PEOU, however, low engagement reduces acceptance of AI (Liang et al., 2024; Thao and Thuy, 2023). Societal expectations related elements, such as social influence, instructor support, and facilitating conditions enhances PU and BI through subjective norms, if ethical and trust-related barriers are handled efficiently (Tram, 2025; Ge, 2024; Mahfouz and AbdelMohsen, 2025).

Learning strategies, such as scaffolded revision, verification, and higher-order problem-solving, boost PU and ATU if aligned pedagogically, but negatively influence critical thinking if not planned properly (Yang and Liu, 2024; Thao and Thuy, 2023). However, trust, ethics, privacy, familiarity, and fear of over-reliance may hinder the acceptance of AI as mostly current studies remain cross-sectional and tool-specific, call for longitudinal and experimental validation in future (Choung et al., 2022; Acosta-Enriquez et al., 2024). Integration of these four CLT constructs into TAM develops the AI Constructivist Learning Model (AICLM), which offers a practical and English education-centered framework. This framework links acceptance behaviors of students with meaningful pedagogical design and supports interventions such as AI literacy, scaffolded learning, and institutional support for responsible AI acceptance and adoption (Tram, 2025; Liang et al., 2024; Mahfouz and AbdelMohsen, 2025).

## 5 Conclusion

This systematic review concludes that external variables, namely motivation, engagement, societal expectations, and constructivist learning strategies, grounded in CLT dominantly shape students’ attitudes toward AI acceptance in improving English writing. Additionally, this review confirms the relevance of the traditional TAM constructs of PEOU, PU, and attitude with affective aspects of CLT. Findings from 16 empirical studies (2021–2025) indicate that motivation enhances PU, engagement enhances PU and PEOU, societal expectations boost adoption through social influence and facilitating conditions, and learning strategies align AI use with accurate, higher-order learning tasks, therefore, strengthening both PU and ATU. These findings consistently show that students are more willing to adopt AI tools when they perceive them as useful, easy to use, motivating, and aligned with meaningful learning practices. The inclusion of these four variables provides a comprehensive view of AI acceptance in language learning contexts by emphasizing affective aspects, cultural and peer influences, and strategic use. The complex interaction between learner psychology, social context, and pedagogical needs in AI-assisted writing is better understood through the proposed extension of TAM. Based on the reviewed evidence, this implies that teachers should integrate AI tools with guided learning strategies, curriculum designers should combine AI literacy and ethical use into writing curricula, and policymakers should support institutional frameworks that promote responsible AI adoption in education. The findings of this review and AICLM may limit its generalizability across different educational levels and cultural backgrounds, as this review is limited by its dependence on studies published mainly from higher education contexts and within a narrow timeframe. Future research is encouraged to empirically validate the modified AICLM across various backgrounds and explore long-term effects of these external variables on the attitudes of students toward the acceptance of AI tools in English language learning.

## Data Availability

The original contributions presented in the study are included in the article/supplementary material, further inquiries can be directed to the corresponding author.

## References

[ref1] Abd Hadi N. A. Mohamad F. Johar E. M. Kadir Z. A. 2024 Exploring the acceptance of ChatGPT as an assisting tool in academic writing among ESL undergraduate students 8 (10), 2886–2901 Int. J. Res. Innov. Soc. Sci. doi: 10.47772/IJRISS.2024.8100242

[ref2] AbuSahyon A. S. E. Alzyoud A. Alshorman O. Al-Absi B. (2023). AI-driven technology and chatbots as tools for enhancing English language learning in the context of second language acquisition: a review study. Int. J. Membrane Sci. Technol. 10, 1209–1223. doi: 10.15379/ijmst.v10i1.2829

[ref3] Acosta-Enriquez B. G. Vargas C. G. A. P. Jordan O. H. Ballesteros M. A. A. Morales A. E. P. (2024). Exploring attitudes toward ChatGPT among college students: an empirical analysis of cognitive, affective, and behavioral components using path analysis. Comput. Educ. Artif. Intell. 7:100320. doi: 10.1016/j.caeai.2024.100320

[ref4] Adiguzel T. Kaya M. H. Cansu F. K. (2023). Revolutionizing education with AI: exploring the transformative potential of ChatGPT. Contemp. Educ. Technol. 15:ep429. doi: 10.30935/cedtech/13152

[ref5] Alamer A. Lee J. (2021). Language learners’ acceptance of AI-based writing assistants: a UTAUT2 perspective. Comput. Educ. Artif. Intell. 2:100031.

[ref6] Alghamdi A. K. H. Palaiologou I. (2021). The impact of AI-based tools on EFL writing development: a systematic review. Lang. Learn. Technol. 25, 1–20.

[ref9001] Alhumsi M. H. Alshaye R. A. (2021). Applying Technology Acceptance Model to Gauge University Students’ Perceptions of Using Blackboard in Learning Academic Writing. Knowledge Management & E-Learning, 13, 316–333. Available online at: http://www.kmel-journal.org/ojs/index.php/online-publication

[ref7] Almushayt A. (2022). Artificial intelligence in English language learning: a systematic review of empirical studies. Educ. Inf. Technol. 27, 2197–2223. doi: 10.1007/s10639-021-10744-y

[ref8] Alsaedi N. S. Alhumsi M. H. (2024). Saudi undergraduate students’ perceptions of plagiarism: a case of EFL research writing tasks during e-learning sessions. Heliyon 10:e39804. doi: 10.1016/j.heliyon.2024.e39804, 39641084 PMC11617220

[ref9] Awal M. R. Haque M. E. (2025). Revisiting university students’ intention to accept AI-powered chatbot with an integration between TAM and SCT: a south Asian perspective. J. Appl. Res. Higher Educ. 17, 594–608. doi: 10.1108/JARHE-11-2023-0514

[ref10] Ayala-Pazmiño M. F. (2023). Artificial intelligence in education: exploring the potential benefits and risks. 593 Dig. Publish. CEIT 8, 892–899. doi: 10.33386/593dp.2023.3.1827

[ref11] Balta N. (2023). Embracing the future: AI’s transformative potential in educational research. Eur. Educ. Res. 6, 1–2. doi: 10.31757/euer.624

[ref12] Bandura A. (1986). Social foundations of thought and action: a social cognitive theory. Hoboken, NJ: Prentice Hall.

[ref13] Binns R. Veale M. Van Kleek M. Shadbolt N. 2018. “It’s reducing a human being to a percentage”: perceptions of justice in algorithmic decisions. In CHI Conference on Human Factors in Computing Systems. New York, NY ACM Digital Library (1–14).

[ref14] Blake R. (2008). “Distance learning for second and foreign language teaching” in Encyclopedia of language and education (Boston, MA: Springer), 1454–1465.

[ref15] Chapelle C. A. Sauro S. (Eds.) (2017). The handbook of technology and second language teaching and learning. Hoboken, NJ: Wiley-Blackwell.

[ref16] Chiu T. K. Moorhouse B. L. Chai C. S. Ismailov M. (2024). Teacher support and student motivation to learn with artificial intelligence (AI) based chatbot. Interact. Learn. Environ. 32, 3240–3256. doi: 10.1080/10494820.2023.2172044

[ref17] Choung H. David P. Ross A. (2022). Trust and ethics in AI. AI Soc. 38, 733–745. doi: 10.1007/s00146-022-01473-4, 41467166

[ref18] Davis F. D. (1989). Perceived usefulness, perceived ease of use, and user acceptance of information technology. MIS Q. 13, 319–340. doi: 10.2307/249008

[ref19] Deci E. L. Ryan R. M. (1985). Intrinsic motivation and self-determination in human behavior. New York, NY: Plenum.

[ref20] Deci E. L. Ryan R. M. (2000). The “what” and “why” of goal pursuits: human needs and the self-determination of behavior. Psychol. Inq. 11, 227–268. doi: 10.1207/S15327965PLI1104_01

[ref21] Divekar R. R. Drozdal J. Chabot S. Zhou Y. Su H. Chen Y. . (2021). Foreign language acquisition via artificial intelligence and extended reality: design and evaluation. Comput. Assist. Lang. Learn. 35, 2332–2360. doi: 10.1080/09588221.2021.1879162, 41307611

[ref22] Donthu N. Kumar S. Mukherjee D. Pandey N. Lim W. M. (2021). How to conduct a bibliometric analysis: an overview and guidelines. J. Bus. Res. 133, 285–296. doi: 10.1016/j.jbusres.2021.04.070

[ref23] Farooq M. Buzdar H. Q. Yen Y. Y. Bakhsh A. (2024). Integrating AI in sustainable writing: an empirical investigation of the technology acceptance model in Asian social sciences. J. Logist. Inf. Serv. Sci. 11, 324–338. doi: 10.33168/JLISS.2024.0321

[ref24] Farooq S. Farooq B. Basheer S. Walia S. (2024). “Balancing environmental sustainability and privacy ethical dilemmas in AI-enabled smart cities” in Exploring ethical dimensions of environmental sustainability and use of AI (Palmdale, PA: IGI Global Scientific Publishing), 263–286.

[ref25] Fishbein M. Ajzen I. (1975). Belief, attitude, intention, and behavior: an introduction to theory and research. Reading, MA: Addison-Wesley.

[ref26] Florea A. M. Radu S. 2019, Artificial intelligence and education. In 2019 22nd International Conference on Control Systems and Computer Science (CSCS) (381–382). Piscataway, NJ: IEEE.

[ref27] Fredricks J. A. Blumenfeld P. C. Paris A. H. (2004). School engagement: potential of the concept, state of the evidence. Rev. Educ. Res. 74, 59–109. doi: 10.3102/00346543074001059

[ref28] Ge T. (2024). Assessing the acceptance and utilization of ChatGPT by Chinese university students in English writing education. Int. J. Learn. Teach. 10, 166–170. doi: 10.18178/ijlt.10.1.166-170

[ref29] Geldbach E. (2023). Redefining the teacher's role in education through artificial general intelligence (AGI). doi: 10.31219/osf.io/b83ps

[ref30] Guan C. Mou J. Jiang Z. (2020). Artificial intelligence innovation in education: a twenty-year data-driven historical analysis. Int. J. Innov. Stud. 4, 134–147. doi: 10.1016/j.ijis.2020.09.001

[ref31] Herrington J. Reeves T. C. Oliver R. (2010). A guide to authentic e-learning. Abingdon: Routledge.

[ref32] Higgins J. P. T. Thomas J. Chandler J. Cumpston M. Li T. Page M. J. . (2022). Cochrane handbook for systematic reviews of interventions. London: Cochrane. 10.1002/14651858.ED000142 PMC10284251 31643080

[ref33] Hu J. Xiao W. (2025). What are the influencing factors of online learning engagement? A systematic literature review. Front. Psychol. 16:1542652. doi: 10.3389/fpsyg.2025.1542652, 40166394 PMC11955628

[ref34] Huang H. M. Liaw S. S. (2005). Exploring user’s attitudes and intentions toward the web as a survey tool. Comput. Human Behav. 21, 729–743. doi: 10.1016/j.chb.2004.02.020

[ref35] Ifinedo P. (2017). Technology acceptance by health professionals in Canada: an analysis with a modified UTAUT model. Stud. Health Technol. Inform. 245, 581–585.29295162

[ref36] Jonassen D. H. (1999). “Designing constructivist learning environments” in Instructional-design theories and models: a new paradigm of instructional theory. ed. Reigeluth C. M. (University Park, TX: Pennsylvania State University).

[ref9002] Jones E. A. Hoffman S. (1995). National assessment of College student learning: identifying College graduates’ essential skills in writing, speech and listening, and critical thinking: final project report. Department of Education Office of Educational.

[ref37] Kannan J. Munday P. (2018). “New trends in second language learning and teaching through the lens of ICT, networked learning, and artificial intelligence” in Vías de transformación en la enseñanza de lenguas con mediación tecnológica. Círculo de Lingüística Aplicada a la Comunicación. eds. Fernández Juncal C. Hernández Muñoz N. (Fairfield, CT: Sacred Heart University).

[ref38] Kelkar S. (2022). Between AI and learning science: the evolution and commercialization of intelligent tutoring systems. IEEE Ann. Hist. Comput. 44, 20–30. doi: 10.1109/MAHC.2022.3143816

[ref39] Keller J. M. (2008). First principles of motivation to learn and e-learning. Dist. Educ. 29, 175–185. doi: 10.1080/01587910802154970

[ref40] Khan R. Qamar M. T. Ansari M. S. Yasmeen J. (2025). Enhancing or impairing? Exploring Indian EFL learners’ academic writing narratives with ChatGPT. Cogent Educ. 12:2514329. doi: 10.1080/2331186X.2025.2514329

[ref41] Khanfar A. A. Kiani Mavi R. Iranmanesh M. Gengatharen D. (2025). Factors influencing the adoption of artificial intelligence systems: a systematic literature review. Manag. Decis. 63, 3727–3755. doi: 10.1108/MD-05-2023-0838

[ref42] Kohnke L. Moorhouse B. L. Zou D. (2023). ChatGPT for language teaching and learning. RELC J. 54, 537–550. doi: 10.1177/00336882231162868

[ref43] Kot S. O. Nykyporets S. S. (2024). “Utilization of artificial intelligence in enhancing English language proficiency in tertiary education” in Science and education in the third millennium Lublin, Poland: Information technology, education, law, psychology, social sphere, management.

[ref44] Landis J. R. Koch G. G. (1977). An application of hierarchical kappa-type statistics in the assessment of majority agreement among multiple observers. Biometrics 33, 363–374. doi: 10.2307/2529786, 884196

[ref45] Leli L. (2020). Analysis of coherence and cohesion on students’ academic writing: a case study at the 3rd year students at English education program. Alsuna J. Arabic Engl. Lang. 3, 74–82. doi: 10.31538/alsuna.v3i2.980

[ref46] Li J. Wang L. (2022). Culture and the adoption of AI in language learning: a cross-cultural study. Educ. Technol. Res. Dev. 70, 1503–1522.

[ref47] Liang J. Huang F. Teo T. (2024). Understanding Chinese university EFL learners’ perceptions of AI in English writing. Int. J. Comput. Assist. Lang. Learn. Teach. 14, 1–16. doi: 10.4018/IJCALLT.358918

[ref48] Liberati A. Altman D. G. Tetzlaff J. Mulrow C. Gøtzsche P. C. Ioannidis J. P. . (2009). The PRISMA statement for reporting systematic reviews and meta-analyses of studies that evaluate healthcare interventions: explanation and elaboration. BMJ 339:b2700. doi: 10.1136/bmj.b2700, 19622552 PMC2714672

[ref49] Ma Z. Q. Cui X. Liu W. P. Tu Y. F. Hwang G. J. (2025). ChatGPT-assisted collaborative argumentation. Educ. Technol. Soc. 28, 133–150. doi: 10.30191/ETS.202507_28(3).SP09

[ref50] Mahfouz I. M. AbdelMohsen M. M. (2025). Investigating college students’ attitudes and perceptions of using ChatGPT in writing language essays. Arab World English J. (AWEJ) Spec. Issue Artif. Intell., 21–39. doi: 10.24093/awej/AI.2

[ref51] Mamédio C. Santos C. Andrucioli De Mattos Pimenta C. Roberto M. Nobre C. (2009). The pico strategy for the research question construction and evidence search. Rev. Lat. Am. Enfermagem. 15, 508–511. doi: 10.1590/s0104-11692007000300023 17653438

[ref52] Mansoor H. S. Khan A. B. Johan Syah M. F. (2025b). ESL undergraduates’ views on the collaborative learning approach and its relationship with their attitudes toward learning English. Forum Linguist. Stud. 7, 516–530. doi: 10.30564/fls.v7i6.9391

[ref53] Mansoor H. S. Sumardjoko B. Sutopo A. (2025a). Attitudes of Pakistani undergraduate ESL students toward artificial intelligence in improving English writing skills. World J. Engl. Lang. 15:369. doi: 10.5430/wjel.v15n6p369 PMC12868173 41646630

[ref54] Mansoor H. S. Sumardjoko B. Sutopo A. Prayitno H. J. Khan A. B. (2025c). Exploring the views of Pakistani ESL teachers about differentiated instruction in English language teaching. Int. J. Engl. Lang. Lit. Stud. 14, 115–122. doi: 10.55493/5019.v14i2.5441

[ref55] McCalla G. (2023). “The history of artificial intelligence in education–the first quarter century” in Handbook of artificial intelligence in education (Cheltenham: Edward Elgar Publishing), 10–29.

[ref56] McHugh M. L. (2012). Interrater reliability: the kappa statistic. Biochem. Med. 22, 276–282. doi: 10.11613/BM.2012.031, 23092060 PMC3900052

[ref57] Mohammed S. H. Kinyo L. (2025). Differences between Arabic and English medium school learners in terms of E-learning technology acceptance and use from the aspect of the constructivist learning approach. Turk. Online J. Distance Educ. 26, 256–287. doi: 10.17718/tojde.1550754

[ref58] Moher D. Liberati A. Tetzlaff J. Altman D. G. (2009). Preferred reporting items for systematic reviews and meta-analyses: the PRISMA statement. PLoS Med. 6:e1000097. doi: 10.1371/journal.pmed.1000097, 19621072 PMC2707599

[ref59] Nguyen P. H. Dieu N. B. (2024). An investigation into third-year ELT students’ perceptions of using Chatgpt as an Ai writing-assistant tool: a case study in Vietnam. Int. J. Arts Humanit. Soc. Sci. Stud. 9, 41–55.

[ref60] Nicol D. Macfarlane-Dick D. (2006). Formative assessment and self-regulated learning: a model and seven principles of good feedback practice. Stud. High. Educ. 31, 199–218. doi: 10.1080/03075070600572090

[ref61] Nightingale A. (2009). A guide to systematic literature reviews. Surgery (Oxf.) 27, 381–384. doi: 10.1016/j.mpsur.2009.07.005

[ref62] Oxford R. L. (2011). Strategies for learning a second or foreign language. Lang. Teach. 44, 167–180. doi: 10.1017/S0261444810000492

[ref63] Page M. J. McKenzie J. E. Bossuyt P. M. Boutron I. Hoffmann T. C. Mulrow C. D. . (2021). The PRISMA 2020 statement: an updated guideline for reporting systematic reviews. BMJ 372:n71. doi: 10.1136/bmj.n71, 33782057 PMC8005924

[ref64] Park S. Y. (2009). An analysis of the technology acceptance model in understanding university students’ behavioral intention to use e-learning. Educ. Technol. Soc. 12, 150–162.

[ref65] Perianes-Rodriguez A. Waltman L. Van Eck N. J. (2016). Constructing bibliometric networks: a comparison between full and fractional counting. J. Informetr. 10, 1178–1195. doi: 10.1016/j.joi.2016.10.006

[ref66] Pokrivčáková S. (2019). Preparing teachers for the application of AI-powered technologies in foreign language education. J. Lang. Cult. Educ. 7, 135–153. doi: 10.2478/jolace-2019-0025

[ref67] Ramzan M. Mushtaq A. Ashraf Z. (2023). Evacuation of difficulties and challenges for academic writing in ESL learning. Univ. Chitral J. Linguist. Lit. 7, 42–49. doi: 10.33195/maxskq26

[ref68] Reeve J. (2012). “A self-determination theory perspective on student engagement” in Handbook of research on student engagement (Boston, MA: Springer), 149–172.

[ref69] Rosen L. D. Weil M. M. (1995). Computer availability, computer experience and technophobia among public school teachers. Comput. Human Behav. 11, 9–31. doi: 10.1016/0747-5632(94)00018-D

[ref70] Salam U. (2025). The integration of ChatGPT in English for foreign language course: elevating AI writing assistant acceptance. Comput. Sch. 42, 145–165. doi: 10.1080/07380569.2024.2446239

[ref71] Scherer R. (2025). Is the technology acceptance model just old wine in new wineskins? Exploring issues for further model development. J. Univ. Teach. Learn. Pract. 22, 1–15. doi: 10.53761/var55x96

[ref72] Schunk D. H. Pintrich P. R. Meece J. L. (2014). Motivation in education: theory, research, and applications. Boston: Pearson Higher Ed.

[ref73] Shadiev R. Wang X. (2022). A review of research on technology-supported language learning and 21st century skills. Front. Psychol. 13:897689. doi: 10.3389/fpsyg.2022.897689, 35874353 PMC9302567

[ref9003] Shi Y. Wang X. Borhan M. S. Young J. Newman D. Berg E. (2021). A review on meat quality evaluation methods based on non-destructive computer vision and artificial intelligence technologies. Food science of animal resources, 41:563. doi: 10.5851/kosfa.2021.e25 34291208 PMC8277176

[ref74] Teo T. Noyes J. (2011). Exploring teacher acceptance of technology: a structural equation modeling approach. Comput. Educ. 57, 2432–2440.

[ref75] Teo H. H. Wang X. Wei K. K. Sia C. L. Lee M. K. (2006). Organizational learning capacity and attitude toward complex technological innovations: an empirical study. J. Am. Soc. Inf. Sci. Technol. 57, 264–279. doi: 10.1002/asi.20275

[ref76] Thao L. Thuy P. (2023). Exploring the impacts of ChatGPT in EFL writing: student perceptions of opportunities and challenges in Vietnamese higher education. Kognisi: Jurnal Ilmu Keguruan 1, 107–124. doi: 10.59698/kognisi.v1i2.175

[ref77] Tlili A. Zhang J. Papamitsiou Z. Manske S. Huang R. Kinshuk . (2021). Towards utilising emerging technologies to address the challenges of using open educational resources: a vision of the future. Educ. Technol. Res. Dev. 69, 515–532. doi: 10.1007/s11423-021-09993-4

[ref78] Tram N. H. M. (2025). Unveiling the drivers of AI integration among language teachers: integrating UTAUT and AI-TPACK. Comput. Sch. 42, 100–120. doi: 10.1080/07380569.2024.2441155

[ref79] UNESCO (2021). AI and education: guidance for policy-makers. Paris: United Nations Educational, Scientific and Cultural Organization.

[ref80] Ushioda E. (2011). Language learning motivation, self and identity: current theoretical perspectives. Comput. Assist. Lang. Learn. 24, 199–210. doi: 10.1080/09588221.2010.538701

[ref81] Van Eck N. Waltman L. (2010). Software survey: VOSviewer, a computer program for bibliometric mapping. Scientometrics 84, 523–538. doi: 10.1007/s11192-009-0146-3, 20585380 PMC2883932

[ref82] Venkatesh V. Bala H. (2008). Technology acceptance model 3 and a research agenda on interventions. Decis. Sci. 39, 273–315. doi: 10.1111/j.1540-5915.2008.00192.x

[ref83] Venkatesh V. Davis F. D. (2000). A theoretical extension of the technology acceptance model: four longitudinal field studies. Manag. Sci. 46, 186–204. doi: 10.1287/mnsc.46.2.186.11926, 19642375

[ref84] Venkatesh V. Morris M. G. Davis G. B. Davis F. D. (2003). User acceptance of information technology: toward a unified view. MIS Q. 27, 425–478.

[ref85] Vorobyeva K. I. Belous S. Savchenko N. V. Smirnova L. M. Nikitina S. A. Zhdanov S. P. (2025). Personalized learning through AI: pedagogical approaches and critical insights. Contemp. Educ. Technol. 17:ep574. doi: 10.30935/cedtech/16108

[ref86] Vygotsky L. S. (1978). Mind in society: the development of higher psychological processes. Cambridge, MA: Harvard University Press.

[ref87] Wang Q. (2025). EFL learners’ motivation and acceptance of using large language models in English academic writing: an extension of the UTAUT model. Front. Psychol. 15:1514545. doi: 10.3389/fpsyg.2024.1514545, 39902118 PMC11788289

[ref88] Weinstein C. E. Mayer R. E. (1986). “The teaching of learning strategies” in Handbook of research on teaching. ed. Wittrock M. (New York, NY: Macmillan).

[ref89] Wood D. Bruner J. S. Ross G. (1976). The role of tutoring in problem solving. J. Child Psychol. Psychiatry 17, 89–100. doi: 10.1111/j.1469-7610.1976.tb00381.x, 932126

[ref90] Xu Z. (2024). AI in education: enhancing learning experiences and student outcomes. Appl. Comput. Eng. 51, 104–111. doi: 10.54254/2755-2721/51/20241187

[ref91] Yang H. Liu M. (2024). Machine translation use in English academic reading and writing: from the perspective of technology acceptance model. Adv. Eng. Technol. Res. 12, 465–465. doi: 10.56028/aetr.12.1.465.2024

[ref92] Zawacki-Richter O. Marín V. I. Bond M. Gouverneur F. (2019). Systematic review of research on artificial intelligence applications in higher education - where are the educators? Int. J. Educ. Technol. High. Educ. 16:39. doi: 10.1186/s41239-019-0171-0

[ref93] Zimmerman B. J. (2002). Becoming a self-regulated learner: an overview. Theory Pract. 41, 64–70. doi: 10.1207/s15430421tip4102_2

[ref94] Zou W. Man S. S. Hu W. Zhou S. Chan H. S. (2025). Factors influencing the acceptance of industry 4.0 technologies in various sectors: a systematic review and meta-analysis. Appl. Sci. 15:4866. doi: 10.3390/app15094866

[ref95] Zou D. Xie H. Wang F. L. Wong T. L. (2020). Intelligent language learning facilitated by artificial intelligence: a perspective. Comput. Assist. Lang. Learn., 1–22. doi: 10.1080/09588221.2020.1846312

